# Effects of vitrification solution supplemented with platelet-rich plasma in rat ovarian tissue cryopreservation

**DOI:** 10.55730/1300-0144.5694

**Published:** 2023-04-04

**Authors:** Aylin GÖKHAN, Türker ÇAVUŞOĞLU, Kubilay Doğan KILIÇ, Cansın ŞİRİN, Canberk TOMRUK, Gürkan YİĞİTTÜRK, Oytun ERBAŞ, Eser YILDIRIM SÖZMEN, Meral BAKA

**Affiliations:** 1 Department of Histology and Embryology, Faculty of Medicine, Ege University, İzmir, Turkiye; 2 Department of Histology and Embryology, Faculty of Medicine, İzmir Bakırçay University, İzmir, Turkiye; 3 Department of Histology and Embryology, Republic of Turkiye Ministry of Health Samsun Education and Research Hospital, Samsun, Turkiye; 4 Department of Histology and Embryology, Faculty of Medicine, Muğla Sıtkı Koçman University, Muğla, Turkiye; 5 Department of Physiology, Faculty of Medicine, Demiroğlu Bilim University, İstanbul, Turkiye; 6 Department of Medical Biochemistry, Faculty of Medicine, Ege University, İzmir, Turkiye

**Keywords:** Calcium, cryopreservation, platelet-rich plasma, ovary, vitrification

## Abstract

**Background/aim:**

The subject of this study was to investigate the utility of platelet-rich plasma (PRP) in the cryopreservation process to reduce cryodamage and increase tissue viability.

**Materials and methods:**

Twenty-one female Wistar rats were randomly allocated to three groups. In Group 1 (G1), rats were not subjected to vitrification (n = 7). Group 2 (G2) was the vitrification group in which PRP was added to the basic vitrification solution (n = 7). Group 3 (G3) was the vitrification group in which fetal bovine serum was added to the basic vitrification solution (n = 7). Warmed tissues were evaluated with histochemical (HC) and immunohistochemical (IHC) staining, the TUNEL method, immunofluorescence (IF) staining, and biochemical analyses.

**Results:**

The percentages of IHC staining, TUNEL method positivity, and IF staining were significantly higher in G2 compared to both G1 and G3 (P < 0.05). G2 ovaries exhibited a significant increase in both malondialdehyde and catalase values in comparison to G1 (P < 0.05). In HC staining, degenerations in primary and secondary follicles and in ovarian tissue were more common in the PRP-supplemented group. The calcium used in PRP activation was suspected to have increased the degeneration and prevented the possible positive effects of PRP.

**Conclusion:**

To the best of our knowledge, PRP-supplemented vitrification solution was used for the first time in the literature in this study in whole rat ovarian tissue vitrification. If PRP is to be used as a component in vitrification solution for rat ovarian tissue, the use of lower amounts of calcium or different methods in PRP activation, or the use of nonactivated PRP, should be considered from the beginning.

## 1. Introduction

Sperm, oocyte, or embryo cryopreservation is still the most widely used technique in infertility applications. However, these practices may not yield rapid results in cases where fertility preservation is desired to be initiated immediately [1]. Ovarian tissue cryopreservation emerges as an alternative feasible solution for patients who need urgent chemotherapy or radiotherapy and do not have time for oocyte retrieval procedures or for coping with hormonal induction complications, who are at risk of premature ovarian insufficiency due to nononcological diseases or benign conditions, who cannot undergo transvaginal oocyte retrieval at a prepubertal/pubertal/adult age, or who are not ready to have a child due to personal reasons but wish to preserve their fertility [2]. Currently, ovarian tissue cryopreservation is assumed to be the only technique that can be offered to patients with prepubertal cancer. It is a safe fertility-preserving approach that should be considered for every patient who will receive gonadotoxic therapy for cancer or noncancer reasons [3].

The success of ovarian tissue cryopreservation procedures is influenced by several factors such as the size of the tissue, the freezing-thawing method (slow freezing or vitrification), the type and concentration of the cryoprotectant agents, and the supplements to be added to freezing-thawing solutions (macromolecules, serum, antioxidants, etc.). Most of the live births obtained after ovarian tissue transplantation in humans were achieved in studies using a slow freezing technique. However, vitrification is said to be the most effective method for the cryopreservation of ovarian tissue [4]. Nevertheless, it is essential to conduct studies with distinct animal species to standardize a simple and effective vitrification protocol that allows the cryopreservation of ovarian tissue as a whole.

Ethylene glycol (EG) and dimethyl sulfoxide (DMSO) are the common cryoprotectant agents used in vitrification procedures [5], and they are also the most preferred cryoprotectant agents by researchers. However, these cryoprotectant agents are toxic compounds and there is a tendency toward using cryoprotectants at low concentrations and adding serum compounds/antioxidants to cryoprotectant mixtures to reduce the toxicity. Although cryopreservation solutions are often supplemented with fetal bovine serum (FBS) in research studies, the search for an alternative to FBS continues along with concerns about the use of animal sera [6]. In this regard, the use of platelet-rich plasma (PRP) is attracting attention.

PRP is a centrifuged plasma component that contains multiple growth factors in a hyperphysiological ratio due to its high platelet concentration. PRP stimulates cell proliferation, mesenchymal cell chemotaxis and differentiation, angiogenesis, and total collagen production, and it also partially increases the expression of matrix metalloproteinases and endogenous growth factors [7]. PRP has been tried by different clinicians and the regeneration-enhancing effect of PRP in gynecological disorders has been demonstrated by various application methods, including intraovarian PRP injection [8]. Based on these points, in this study, it was aimed to assess whether supplementing vitrification solution with PRP could improve cryopreservation outcomes. We hypothesized that our results would contribute to future studies on tissue transplantation, which is mentioned as an ideal method for the evaluation of viability and endocrine functions after cryopreservation, as well as to tissue banking research.

## 2. Materials and methods

### 2.1. Ethical statement

This study was carried out with the approval of Ege University’s Animal Experiments Local Ethics Committee (date 28/02/2018, #2018-003). All experimental animals were provided by Ege University’s Laboratory Animals Application and Research Center and all experiments were carried out in accordance with the guidelines confirmed by the National Institutes of Health (NIH Publications No. 8023, revised 1978) and the Local Ethics Committee.

### 2.2. Animals and experimental design

Twenty-one female Wistar rats, 8–12 weeks old, were randomly allocated to groups with seven animals in each group. Group 1 (G1, control) was not subjected to vitrification (n = 7). Group 2 [G2, PRP (+)] was the vitrification group in which PRP was added to the basic vitrification solution (n = 7). Group 3 [G3, PRP (−)] was the vitrification group in which FBS was added to the basic vitrification solution (n = 7). The basic vitrification solution was composed of EG, DMSO, and sucrose as the most preferred cryoprotectant agents. Blood samples of 6 mL, used to prepare PRP, were harvested by cardiac puncture separately from each rat in G2. All animals were anesthetized with a combination of 10 mg/kg xylazine (Bayer) and 80 mg/kg ketamine hydrochloride (Pfizer) and euthanized by cervical dislocation after the surgical procedure.

After oophorectomy, the left ovaries of the rats of the control group were placed in the fixative solution for tissue processing and the right ovaries were transferred directly to the biochemistry laboratory. In the vitrification groups, the ovaries from both sides were transferred to the laboratory within the base medium after removal. Cold-chain conditions were ensured during transportation. From fresh and vitrified/warmed tissues, the left-sided ovaries were subjected to microscopic analyses including histochemical (HC) and immunohistochemical (IHC) staining, terminal deoxynucleotidyl transferase dUTP nick end labeling (TUNEL) testing, and immunofluorescence (IF) staining, and the right-sided ovaries were subjected to biochemical analyses including measurements of the tissue levels of myeloperoxidase (MPO), malondialdehyde (MDA), and catalase (CAT).

### 2.3. Preparation of PRP

The method of Chen et al. [9] was modified and applied in the PRP preparation process. Briefly, 6 mL of blood was harvested via cardiac puncture from each rat in G2 under general anesthesia. Each of the individual blood samples was immediately mixed with 3.2% sodium citrate as an anticoagulant at a ratio of 9:1 blood to citrate. The first centrifugation process at 220 × *g* spin (20 min) at 4°C separated the whole blood into three layers. The supernatant containing the top layer (plasma) and the middle layer (the buffy coat devoid of red blood cells) was transferred to empty tubes. The new tubes were spun at 480 × *g* (20 min) at 4 °C. The second centrifugation separated the platelet-poor plasma at the top and the PRP at the bottom. The PRP was activated with a 10% (w/v) calcium chloride solution (containing approximately 0.46 M of (CaCl_2_.6H_2_O) at a ratio of 10:1. Finally, CaCl_2_-activated PRP was used as the vitrification solution content.

### 2.4. Cryopreservation procedure

#### 2.4.1. Preparation of solutions

The study model of He et al. [10] was modified and applied in the preparation of base medium, equilibration medium, vitrification solution, warming solution, washing medium, and incubation medium. Media compositions are indicated in Table 1. The list of chemicals and manufacturers is given in the Supplementary File (Section I). The ovaries were exposed to the solutions at room temperature.

#### 2.4.2. Vitrification of ovaries

Ovarian tissues were removed from the base medium and transferred to the equilibration medium. After 15 min in the equilibration medium, G2 ovaries were transferred to vitrification solution number one (VS-1) and G3 ovaries to vitrification solution number two (VS-2). After 2 min in the vitrification solution, the ovarian tissues were placed into cryovials with minimal vitrification solution remaining. Following this process, cryovials with ovarian tissues inside were plunged directly into liquid nitrogen at −196 °C and stored for 1 week.

#### 2.4.3. Warming of ovaries

At the end of 1 week, the ovaries were removed from the storage tank. The ovarian tissues were exposed to warming solutions containing decreasing sucrose concentrations. After sequential rinsing for 5 min in each of the warming solutions (WS-1, WS-2, and WS-3, respectively), the ovaries were washed twice with the washing medium and then incubated for 10 min in the incubation medium.

## 2.5. Microscopic analysis

Fresh and vitrified-warmed ovarian tissues were fixed for 24 h in 4% paraformaldehyde. The tissues were embedded in paraffin blocks following routine tissue processing and sectioned at 5 μm with a microtome (Leica RM 2145). Sections stained with HC and IHC staining, TUNEL testing, and IF staining were then evaluated. All slides were photographed with an Olympus DP72 (Tokyo, Japan) digital camera mounted on an Olympus BX51 (Tokyo, Japan) microscope.

### 2.5.1. HC staining and follicle counting

The histomorphological evaluation of the ovaries was performed on sections that were routinely stained with hematoxylin and eosin (H&E), sections stained with periodic acid–Schiff (PAS) in line with the literature, and sections stained with Mallory trichrome (MT) (Bio-Optica Milano S.p.A., Milan, Italy) according to the manufacturer’s instructions. During serial sectioning, the probability of consecutive counting of the same follicle was reduced by both setting aside one slide in ten and including only the follicles with visible nuclei for the evaluation. Each of three investigators independently reviewed a slide that was selected randomly. The differentiation of ovarian follicles was performed as primordial, primary, secondary, and antral follicles according to the morphological criteria of previous studies [11,12]. Based on the microscopic observation of surrounding pregranulosa or granulosa cells, oocytes with flattened-squamous cells as a single thin layer were defined as primordial follicles. Next, oocytes with a single granulosa cell layer in cuboidal form were classified as primary follicles. Early-stage (developing) primary follicles surrounded by a single row of granulosa cells, most of which were cuboidal and a few in squamous form, were included in the primary follicle class. Finally, categorizing secondary follicles as oocytes containing two or more layers of granulosa cells in cuboidal form without a visible antrum allowed them to be distinguished from antral follicles with a clearly defined antral cavity and cumulus cell layer. The follicles were classified as healthy or degenerated based on criteria such as eosinophilic cytoplasm in the oocyte, pyknosis, and structural integrity including the zona pellucida and granulosa cells [13].

### 2.5.2. IHC staining

The sections were subjected to the routine IHC protocol of our laboratory. Sections were treated with the antibodies of Bcl-2, Bax, caspase-3 (C3), and connexin 43 (Cx-43). The list of antibodies and manufacturers is given in the Supplementary File (Section II). Immunoexpression analyses were performed independently by three observers. Initially, six fields of view per slide were randomly selected at 100× total magnification. The positive immunoreaction percentages were then calculated for each of the six fields at 400× total magnification and finally averaged.

### 2.5.3. TUNEL assay

The TUNEL assay was performed for determining apoptosis using an In Situ Cell Death Detection Kit (Roche, Penzberg, Germany) according to the manufacturer’s instructions. The examination was conducted in the same way as the IHC examination described above.

### 2.5.4. IF staining

C3 IF and TUNEL IF staining procedures were performed according to the manufacturer’s instructions using caspase-3 p17 antibody (D-12) (sc-373730) (Santa Cruz Biotechnology, Santa Cruz, CA, USA) and the ApopTag® Fluorescein in Situ Apoptosis Detection Kit (S7110) (Merck, Darmstadt, Germany), respectively. The examination was conducted in the same way as the method described above, except for the total magnification (200×).

### 2.6. Biochemical analysis

All reagents and analyses were applied in the Department of Medical Biochemistry of our institution in line with the previous literature [14]. The data for MDA measurements and CAT and MPO activities were expressed in nmol/MGPRT, U/MGPRT, and U/MGPRT, respectively.

### 2.7. Statistical analysis

Histochemical data were analyzed with IBM SPSS Statistics 25.0 (IBM Corp., Armonk, NY, USA) in the Department of Biostatistics of our institution. One-way analysis of variance (ANOVA), as a parametric analysis method, was applied to determine whether the data differed according to the independent variables. Variance homogeneity was checked with the Levene test. The F test was used for multiple comparisons for variables with homogeneous variance. The Bonferroni method was used to compare the two post hoc methods after the F test. According to the Levene test, the Welch method was used for multiple comparisons for variables where variances were not homogeneous and the Dunnett T3 test was used as the post hoc method. Biochemical data were analyzed with the Student t-test. Values of P < 0.05 were considered statistically significant in the evaluation of all results.

## 3. Results

### 3.1. HC staining and follicle counting

**Figure 1:**
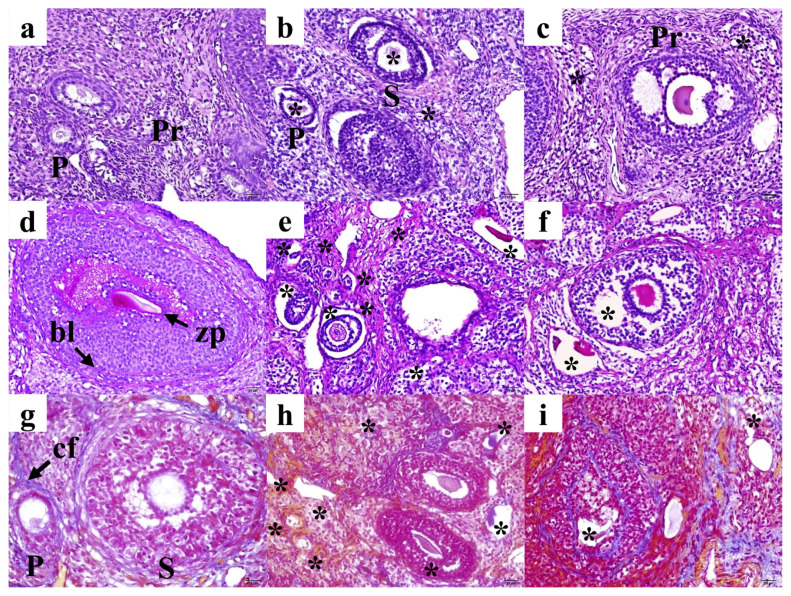
Representative sections from histochemical staining experiments. Study groups are given in columns as G1 (left), G2 (middle), and G3 (right), respectively. Sections are aligned as hematoxylin and eosin (a–c), periodic acid–Schiff (d–f), and Mallory trichrome (g–i), respectively. Scale bar: 20 μm. Total magnification: 400×. **Pr:** Primordial follicle; P: primary follicle; S: secondary follicle; zp: zona pellucida; bl: basal lamina; cf: collagen fibers; asterisk: degeneration.

All HC-stained slides, including staining by H&E (Figures 1a–1c), PAS (Figures 1d–1f), and MT (Figures 1g–1i), were evaluated for histomorphological changes and are presented in Figure 1 as representative images. The histomorphological evaluation of the ovaries included the germinal epithelium, the tunica albuginea, the stroma, the cortex, the medulla, the vessels, the corpora lutea, and the different cellular components, i.e., the oocytes, follicles, and stromal cells.

The control group showed a normal histological appearance (Figures 1a, 1d, and 1g), and both vitrification groups showed deteriorations compared to the control. In particular, the vitrification group with PRP (Figures 1b, 1e, and 1h for G2) was more damaged than the group without PRP (Figures 1c, 1f, and 1i for G3). A more detailed interpretation is available in the Supplementary File (Section III).

The follicular examination included the evaluation of only the follicles that were predicted to be most likely to survive in any cryopreserved ovary [15]. Although primordial follicles are a good indicator of ovarian reserve and toxicology reports are expected to include a qualitative assessment of these follicles [16], they were not included in the follicle counting in this study because the primordial follicles of all groups showed similar morphology (Figure 1). Therefore, counting was performed on small follicles in the growth phase, excluding primordial follicles, which included primary and secondary follicles.

Follicular degenerations were prominent in primary and secondary follicles in both experimental groups (Figures 1b, 1e, and 1h for G2 and 1c, 1f, and 1i for G3). However, G2 showed a significant increase in the percentage of degenerated primary follicles compared to the control (P < 0.05). Statistical analysis of the follicle counts is given in Table 2.

### 3.2. IHC staining, TUNEL assay, and IF staining

**Figure 2:**
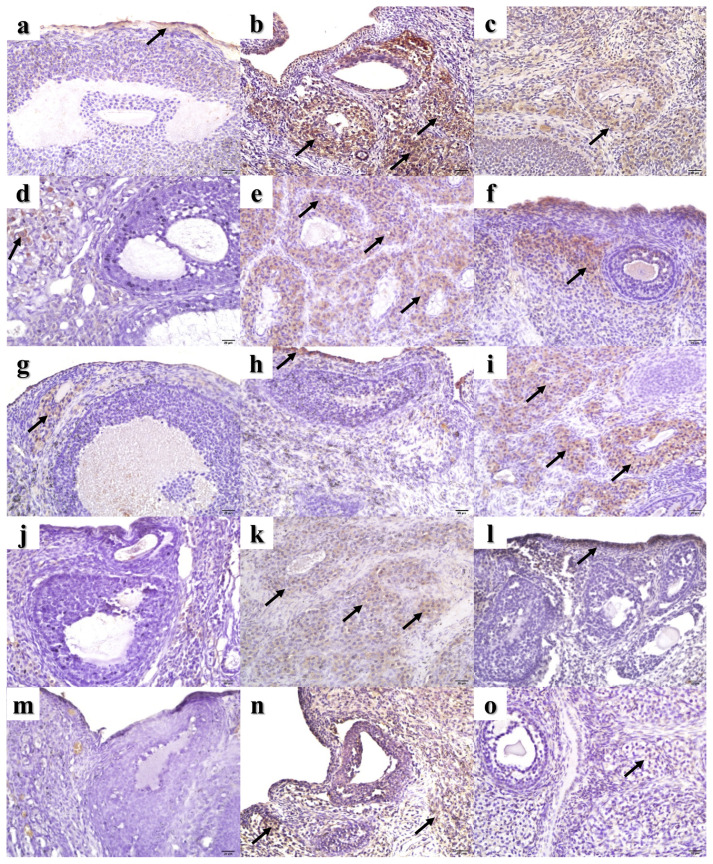
Representative sections from IHC staining and TUNEL assay. Study groups are given in columns as G1 (left), G2 (middle), and G3 (right), respectively. Sections are aligned as Cx-43 (a–c), Bax (d–f), Bcl-2 (g–i), C3 (j–l), and TUNEL (m–o) from top to bottom, respectively. Immunoexpression is indicated with black arrow. Scale bar: 20 μm. Total magnification: 400×

**Figure 3:**
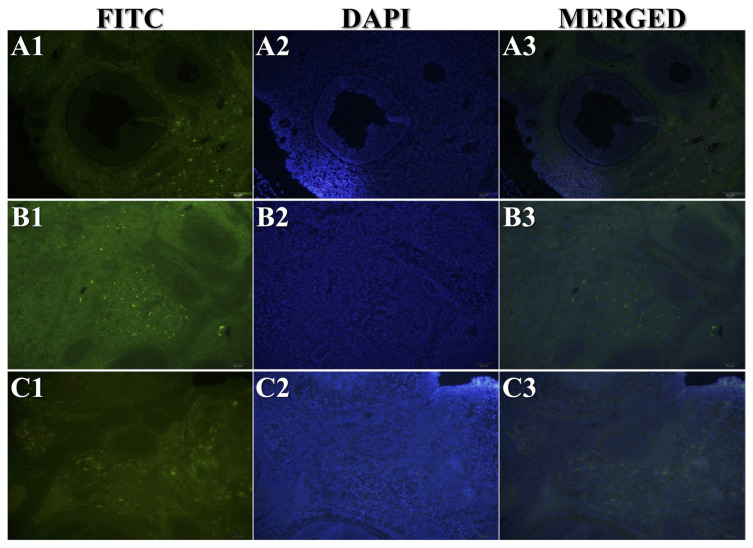
Representative sections from IF staining of C3. Study groups are numbered as G1 (A1–A3), G2 (B1–B3), and G3 (C1–C3), respectively. Total magnification: 200×.

**Figure 4:**
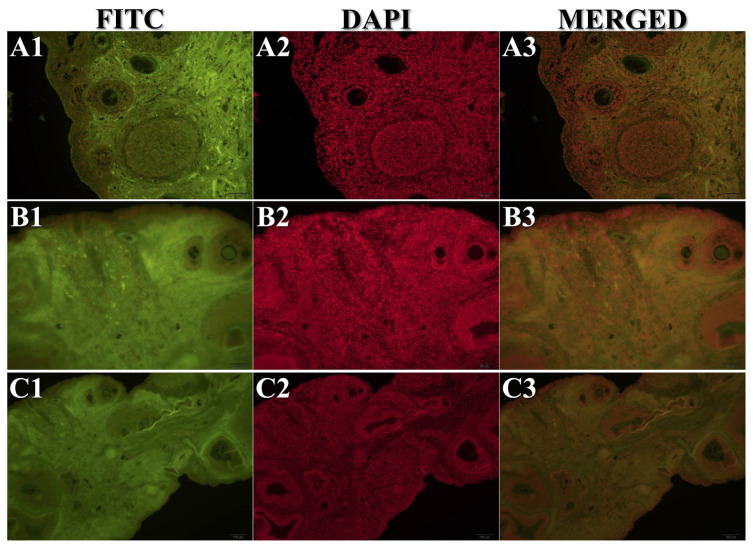
Representative sections from IF staining of TUNEL. Study groups are numbered as G1 (A1–A3), G2 (B1–B3), and G3 (C1–C3), respectively. Total magnification: 200×.

**Figure 5:**
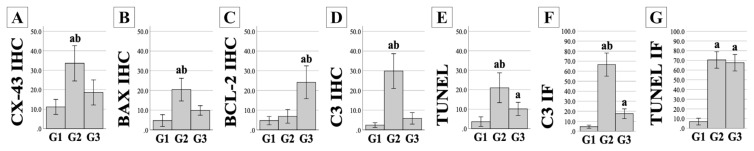
Statistical analysis of immunoexpression results. Vertical axes indicate the results from IHC staining of Cx-43 (A), Bax (B), Bcl-2 (C), and C3 (D); TUNEL assay (E) and IF staining of C3 (F); and IF staining of TUNEL (G). Horizontal axes indicate the study groups, given as G1–G3. All data are given as a percentage (%) with mean ± SD values. Error bars: 95% CI. ^a^: Significant compared to G1 (P < 0.05). ^b^: Significant in the comparison of G2 and G3 (P < 0.05).

All stained slides obtained from the experimental animals were evaluated for immunopositivity. Cx-43 (Figures 2a–2c), Bax (Figures 2d–2f), Bcl-2 (Figures 2g–2i), and C3 (Figures 2j–2l) IHC staining results, TUNEL analysis results (Figures 2m–2o), and C3 IF (Figure 3) and TUNEL IF (Figure 4) staining results are presented as representative images. Percentage values (%) of immunoexpressions from all stained slides were statistically compared between groups (Figure 5).

The percentage of immunostaining with Cx-43 IHC (Figures 2b and 5A), Bax IHC (Figures 2e and 5B), C3 IHC (Figures 2k and 5D), TUNEL analysis (Figures 2n and 5E), and C3 IF (Figures 3B and 5F) was significantly higher in G2 compared to both the control group and G3 (P < 0.05). In addition, the TUNEL IF score of G2 (Figures 4B and 5G) was also significantly higher than that of the control group (P < 0.05).

Bcl-2 immunolabeling results were significantly higher in G3 (Figure 2i and Figure 5C) compared to both the control group and G2 (P < 0.05). Moreover, TUNEL analysis results (Figures 2o and 5E) and the C3 IF (Figures 3C and 5F) and TUNEL IF (Figures 4C and 5G) scores of G3 were significantly higher than those of the control group (P < 0.05). A more detailed interpretation is available in the Supplementary File (Section IV).

### 3.3. Biochemical analysis

G2 ovaries exhibited a significant increase in both MDA and CAT values in comparison to G1 (P < 0.05). G3 ovaries exhibited a significant increase in CAT values in comparison to G1 (P < 0.05). No significant difference between groups was detected in MPO values (P > 0.05). Statistical analysis of the biochemical study results is given in Table 3.

## 4. Discussion

Ovarian tissue vitrification is known to promote an atretic process in most follicles, except primordial follicles, which are known to better tolerate cryopreservation injury due to their unique structure [17]. Consistent with past studies [18], in the current study we also showed that the primordial follicles were mostly well protected in all study groups. However, Oktem et al. detected a significant reduction in primordial follicles and morphological abnormalities in preantral and antral follicles as a result of vitrification of the human ovary [19].

Increased rates of degeneration in both primary and secondary follicles, which were prominent in G2 of the current study, have been reported in previous studies. One of those studies was particularly important as it revealed that the vitrification method applied for rat whole ovarian tissue cryopreservation had a serious lethal effect on the follicles [20]. Severe damage to primary and secondary follicles was also reported in vitrified human, pig, and bovine ovaries, but, in contrast to our findings, the primordial follicles were not preserved [13]. Vitrification-induced damage shown in granulosa cells and oocytes was also similar to our results [21]. On the contrary, there are also reports pointing to preserved granulosa cell morphology [22]. Nevertheless, most studies have agreed that vacuolization and loss of integrity in the follicles and reductions in follicle counts that occur after exposure to vitrification solution are due to osmotic stress, cryoprotectant agent toxicity, or severe cellular damage including mitochondrial deformity [23].

Reactive oxygen species and oxidative stress initiate lipid peroxidation, one of the mechanisms of tissue damage, and MDA is one of the main products released from lipid peroxidation [24]. Thus, it is one of the most highly preferred parameters in clinical studies for the biochemical determination of oxidative stress. The biochemical analysis results obtained in our study were consistent with the literature and supported the histological results we obtained. MDA values revealed as a result of tissue damage were significantly increased in G2 compared to the other groups.

The unexpected increase in apoptotic markers and degenerations in G2 prompted us to investigate the cause, and we suspected that the CaCl_2_ we used to activate the PRP might have provoked this cellular damage. The concentration of CaCl_2_ used in this experiment might have been too high for the rat ovarian tissue vitrification method. A serious loss in live cells with the addition of CaCl_2_ to culture conditions has been reported [25]. Our determinations were similarly expressed in a past study that mentioned the importance of the Ca^2+^ ion in both the release of proapoptotic proteins and the regulation of mitochondrial morphology [26].

Regardless of whether PRP was added or not, intracellular calcium was increased in vitrified rat oocytes with the effect of vitrification itself (i.e., exposure to low temperatures during the process) [27], and it also increased in mouse oocytes with the effect of DMSO and EG [28]. Moreover, vitrification also increased both cytoplasmic free calcium ions ([Ca^2+^]i) and mitochondrial calcium (mCa^2+^) in cryopreserved bovine oocytes, especially when DMSO was used [29]. Excess mCa^2+^ is important because mCa^2+^ overload was reported to cause activation in the opening of membrane permeability transition pores, resulting in mitochondrial membrane rupture. Cryopreserved embryos, oocytes, and sperm were also associated with a reduction in mitochondrial membrane potential (ΔΨm) and ATP content, which are markers of mitochondrial function, and vitrification significantly increased the percentage of TUNEL-positive oocytes due to low ΔΨm values [30]. The loss of ΔΨm can release proteins in the intermembrane space, such as cytochrome c, and then activate the caspase cascade that prompts the apoptotic program [29]. Calcium chelators [29,31], purinergic receptor modulators [32], and calcium-free media [33] have been suggested to protect mitochondrial functions and maintain ΔΨm, thus reducing the deleterious effects of cryopreservation.

The excess of granulosa cells showing a positive TUNEL staining pattern in G2 compared to G3 was associated with a greater abundance of cryodamaged cholesterol molecules in steroid-producing cells, such as granulosa cells. We attributed the less pronounced apoptosis markers in the granulosa cells of G3 to the absence of CaCl_2_ in the vitrification solution of G3. We further hypothesized that the lesser presence of apoptosis in interstitial tissue was due to the cholesterol content not being as high as in steroid-producing cells.

In this study, Bax expression was more intense in G2 than G3, especially in granulosa cells and oocytes in follicle structures, and less intense in the stromal area between follicles. These data, supporting the results of TUNEL analyses, led us to the conclusion that vitrification promoted apoptosis in ovarian tissue but was exaggerated in ovarian tissue cryopreservation performed with CaCl_2_-activated PRP. Although the Bcl-2 IHC staining analysis of our study showed an increase in the percentage of expression in G2 compared to G1, the difference was not significant. However, the significant increase in G3 was attributed to the antiapoptotic effect against cellular damage caused by the apoptosis-inducing effect of vitrification. Contrary to what was expected, the lower percentage of expression seen in G2 was associated with an increased apoptotic effect due to the cell damage caused by CaCl_2_ with increased overexpression of Bax. Vitrification created damage to the inner membrane of the mitochondria and vesicular organelles of the cell, which was exaggerated due to the presence of CaCl_2_ in G2. As the cell membranes were damaged during vitrification and therefore extracellular calcium penetrated more into the cell, the apoptotic and inflammatory pathways were exacerbated. Therefore, the antiapoptotic pathways were not able to be effective enough.

On the other hand, our light microscope examinations showed cellular separations and vacuolization in the follicle structure that were linked to the loss of lateral connections between granulosa cells. Cells undergoing apoptosis were seen between the granulosa cells surrounding the oocytes and the cells in the theca layers. The expression levels in Cx-43 IHC staining showed a significant increase in G2 and an insignificant slight increase in G3. Researchers have shown that Cx-43 was translocated to mitochondria, where it induced mitochondrial outer membrane permeabilization with Bax, followed by cytochrome c release and caspase activation [34]. Additionally, that study revealed that the Bax/Bcl-2 ratio increased with Cx-43 expression. Reports indicating the connection between Cx-43 and Bax [35], as well as studies showing that Cx-43-mediated apoptosis is regulated by a decrease in Bcl-2 expression [36], are available in the literature, similar to the findings obtained in our study.

The connexin-based gap junctional intercellular communications and associations between the oocyte and the cumulus cells have fundamental roles in oocyte development and ovarian folliculogenesis [37,38]. The basic units of gap junctions are connexin hemichannels, namely connexons [39]. The connexin subunits come together to form unopposed hemichannels and gap-junctional channels. When connexons on the membranes of two adjacent cells dock head to head with each other, they form an intercellular channel that allows molecular exchange between the opposing cells. The unopposed or undocked or non-junctional connexin hemichannels primarily reside in the plasma membrane in closed form [40]. They may open under certain physiological conditions such as changes in [Ca^2+^]i [41,42], and also under nonphysiological conditions such as increased extracellular Ca^2+^ [40] or vitrification and warming processes [37]. Following a massive opening, the hemichannels turn into toxic pores that allow an uncontrolled exchange of ions and small molecules between the intra- and extracellular environments. This may result in the rapid spread of cell injury, cell death, and apoptosis to neighboring cells [37]. Of note, the uncontrolled opening of hemichannels is suggested to contribute to Ca^2+^ overload that aggravates cellular disfunction [41].

In our study, while there was a significant increase in G2 compared to both G1 and G3 among the findings obtained for Cx-43 expression (P < 0.05), a slight increase was found when G3 and G1 were compared (P > 0.05). Normally, a decrease in Cx-43 expression is expected due to degeneration. The slight increase in G3 was associated with the opening of Cx-43 hemichannels due to the stress caused by vitrification. The significant increase in G2 was associated with both the previously mentioned effects of vitrification and the effects of calcium. It was concluded that cellular damage occurred as a result of a change in pH, metabolic stress, and oxidative stress due to only vitrification in G3, and also vitrification as well as CaCl_2_, which was added to the vitrification solution for PRP activation, in G2. As a result, the hemichannels might have opened after cell damage and excessive amounts of cryoprotectant and CaCl_2_ might have entered the cells through the pores formed in the membranes. This assumption led to the conclusion that vitrification of rat whole ovarian tissue with calcium-activated PRP might have increased the cellular damage and induced excessive apoptosis, as well as causing apoptosis to spread more rapidly to the surrounding cells.

## 5. Conclusion

In this study, for the first time in the literature, whole rat ovaries were vitrified with autologous CaCl_2_-activated PRP-supplemented solution and the ovaries were examined with histochemical and biochemical investigations. Promising results were expected, as PRP contains a large number of bioactive compounds. However, surprisingly, more unexpected findings were observed in the ovaries vitrified with PRP-supplemented solution. Following a literature search, it was hypothesized that the poor results observed with the use of PRP might have been due to the large amount of CaCl_2_ used to activate the PRP. However, since the main purpose of this study was not to examine PRP activation methods, this assumption should be explored with further studies. Nevertheless, it is recommended that researchers considering adding PRP to rat ovarian tissue vitrification solution use a lower amount of CaCl_2_ or a different method for PRP activation, or consider using nonactivated-PRP. Evaluating the utility of PRP with slow cooling may be beneficial to enhance cryopreservation protocols. Preserving the ovaries as small cortical pieces, rather than whole tissue, may yield more successful results.

## Figures and Tables

**Table 1:** Steps and solutions of cryopreservation process.

Steps		Pre-CP		Vitrification		Warming			Post-CP	
Solutions		BM	EM	VS-1	VS-2	WS-1	WS-2	WS-3	WM	IM
Duration		<30 min	15 min	2 min	2 min	5 min	5 min	5 min	2–3 min	10 min
Components	DMEM/F-12	90%		50%	50%	90%	90%	90%		
	FBS	10%								
	BM		80%						100%	
	PRP			10%						
	FBS				10%	10%	10%	10%		
	EG		10%	20%	20%					
	DMSO		10%	20%	20%					
	S			0.5 mol/L	0.5 mol/L	0.5 mol/L	0.25 mol/L	0.125 mol/L		
	AMEM									100%

Ratios (v/v) of the components (left column) of the media (solutions) used in the rat ovarian tissue cryopreservation protocol. Treatment times are indicated. CP: Cryopreservation; BM: base medium; EM: equilibration medium; VS: vitrification solution; WS: warming solution; WM: washing medium; IM: incubation medium; DMEM/F-12: Dulbecco’s modified Eagle’s medium/nutrient mixture F-12; FBS: fetal bovine serum; PRP: platelet-rich plasma; EG: ethylene glycol; DMSO: dimethyl sulfoxide; S: sucrose; AMEM: alpha modification of Eagle’s medium.

**Table 2:** Follicle counts.

	G1	G2	G3
PFH	85.78 ± 11.64	69.84 ± 11.29*	77.70 ± 8.85
PFD	14.21 ± 11.64	30.15 ± 11.29*	22.29 ± 8.85
SFH	65.02 ± 13.8	43.98 ± 22.51	62.48 ± 14.86
SFD	34.97 ± 13.8	56.01 ± 22.51	37.51 ± 14.86

Study groups are given in columns as G1 (left), G2 (middle), G3 (right). Follicle counts are given in rows. All data are given as a percentage (%) with mean ± SD values.

* Significant compared to G1 (P < 0.05).

PFH: Healthy primary follicle, PFD: degenerated primary follicle, SFH: healthy secondary follicle; SFD: degenerated secondary follicle.

**Table 3:** Biochemical study results.

	G1	G2	G3
MDA	1.34 ± 0.50	3.08 ± 1.82*	1.84 ± 0.57
CAT	2.57 ± 0.91	5.67 ± 4.35*	4.33 ± 0.84*
MPO	0.39 ± 0.13	0.77 ± 0.80	0.29 ± 0.10

Study groups are given in columns as G1 (left), G2 (middle), and G3 (right). Analyses are given in rows. All data are given as mean ± SD.

* Significant compared to G1 (P < 0.05).

MDA: Malondialdehyde; CAT: catalase; MPO: myeloperoxidase.

## Appendix

Supplementary file.

### Section I

**Table S1:** List of chemicals used for the preparation of cryopreservation solutions.

Chemical/product	REF/Cat No	Manufacturer
Dulbecco’s Modified Eagle’s Medium/Nutrient Mixture F-12 (DMEM/F-12)	31330-038	Gibco, Thermo Fisher Scientific
Fetal Bovine Serum (FBS)	10270-106	Gibco, Thermo Fisher Scientific
Ethylene Glycol (EG)	324558	Sigma-Aldrich
Dimethyl Sulfoxide (DMSO)	WAK-DMSO-10	WAK-Chemie Medical GmbH
Sucrose (S)	S-9378	Sigma-Aldrich
Alpha Modification of Eagle’s Medium (AMEM)	310-011-CL	Wisent Inc.

### Section II

**Table S2:** List of primary antibodies used for IHC investigation.

Antibody	Class	Reactivity	Host	Dilution	REF/Cat No	Manufacturer
Bcl-2 (N-19)	Polyclonal	Human, mouse, rat,	Rabbit	1:100	Sc-492	Santa Cruz Biotechnology, Santa Cruz, CA, USA
Bax	Polyclonal	Human, mouse, rat	Rabbit	1:100	34260	SAB Signalway Antibody, Baltimore, USA
Caspase-3 (Phospho-Ser150)	Polyclonal	Human, mouse, rat	Rabbit	1:100	12324	SAB Signalway Antibody, Baltimore, USA
Connexin 43	Polyclonal	Human, mouse, rat, dog	Rabbit	1:100	Bs-0651R	Bioss, USA

### Section III

#### Detailed interpretation of HC staining

G1 sections showed a normal histological appearance. Examination of the experimental groups showed that the integrity of the germinal epithelium was impaired in G2 and was similar to those in the control group for G3. Tunica albuginea was impaired in G2 and partially degenerated in G3. In the medullary region and interfollicular stroma, irregular distribution of the collagen fibers and a decrease in the number of fibers were more prominent in G2. In the medullary region, irregularity in cell borders, cellular dissociation, vacuolization, and vascular disruption are more abundant in G2 compared to G3. In G2, there was irregularity and separation in the basal laminae of the germinal epithelium, follicles, and vessels. Dissociation in granulosa cells and vacuolization in follicles were more prominent in G2. Degenerated oocytes that have acidophilic cytoplasm, pyknotic nuclei, condensed/fragmented chromatin, dense PAS-staining, and degenerated follicles at different developmental stages, were more common in G2. Follicular degenerations were seen especially in primary and secondary follicles, and primordial follicles were mostly preserved in both experimental groups. Several follicles, especially in G2, showed significant changes in the zona pellucida configuration and even the zona pellucida had completely separated from the oocyte. The weakening of the connection between oocyte and surrounding cells and the presence of apoptotic cells amongst granulosa cells and theca cells were more prominent in G2.

### Section IV

**Table S3:** Comparison of immunoexpression in IHC staining, TUNEL assay, and IF staining.

	G1	G2	G3
**CX 43**	Mild expression in GE cells, GCs, and stromal cells	Increased expression esp. in follicular GCs, also in GE and stromal area between follicles	Moderate expression, less than G2, esp. notable in GCs and stromal area between follicles; expression in GE was similar as G1
**BAX**	Partial expression in GCs within atretic follicles due to follicular maturation process	Strong expression esp. in oocytes and GCs in follicles; less intense in stromal area between follicles	Moderate expression, more than G1, less than G2
**BCL-2**	Insignificant positivity in a small proportion of fields consist of GE, stromal areas that follicles located, medullary region	Slightly increased expression compared to G1	Increased expression in multiple follicles at different stages compared to G1 and G2
**C3**	Weak expression in GE, some follicles, stromal area between follicles, medullary region, CL; negative in intact oocytes	Strong expression in GCs of follicles at different stages located especially at cortex, and in CLs detected at some sections; increased expression in irregularly distributed GCs of degenerated follicles in stromal area of cortex	Moderate expression in the stromal areas of cortex adjacent to TA and in CLs; weaker expression in the medullary region compared to G2
**TUNEL**	Weak expression in GE, some follicles, medullary region, stromal area and in CLs detected at some sections	Strong expression esp. in stromal area of cortex and in GCs of follicles at different stages located therewithin; intense staining pattern in CL and in areas of medullary region where vascular structures were condensed	Moderate expression with more than G1, less than G2; partial positivity in GCs of some follicles as well as stromal area between follicles and medullary region
**C3 IF**	Poor reaction in follicular structures, stromal area, and medullary region	Positive reaction more than G1 and G3 in cortex stroma and in GCs of follicles at different stages located therewithin, in CL, in medullary region where vascular structures were condensed	Weaker reaction than G2 in cortex stroma and in GCs of follicles at different stages located therewithin, in CL, in medullary region where vascular structures were condensed
**TUNEL IF**	Poor reaction in stroma between follicles, GCs of follicles at different stages, medullary region	Positive reaction more than G1 in interstitial area between follicles in cortex, GCs, CL, and medullary region; slight increase in expression compared to G3	Positive reaction in interstitial area between follicles in cortex, GCs, CL, and medullary region

Study groups are given in columns as G1 (left), G2 (middle), G3 (right). Analyses are given in rows. GE: germinal epithelium, TA: tunica albuginea, GC: granulosa cell, CL: corpus luteum.
